# Translations of Steinhausen's Publications Provide Insight Into Their Contributions to Peripheral Vestibular Neuroscience

**DOI:** 10.3389/fneur.2021.676723

**Published:** 2021-06-04

**Authors:** Hans Straka, Michael G. Paulin, Larry F. Hoffman

**Affiliations:** ^1^Department Biology II, Ludwig-Maximilians-University Munich, Munich, Germany; ^2^Department of Zoology, University of Otago, Dunedin, New Zealand; ^3^Department of Head & Neck Surgery and the Brain Research Institute, David Geffen School of Medicine at University of California at Los Angeles, Los Angeles, CA, United States

**Keywords:** cupula, endolymph, biomechanical model, crista, labyrinth, torsion-pendulum

## Abstract

The quantitative relationship between angular head movement and semicircular canal function is most often referenced to the well-known torsion-pendulum model that predicts cupular displacement from input head acceleration. The foundation of this model can be traced back to Steinhausen's series of papers between 1927 and 1933 whereby he endeavored to document observations of cupular displacements that would directly infer movement of the endolymph resulting from angular rotation. He also was the first to establish the direct relationship between cupular displacement and compensatory eye movements. While the chronology of these findings, with their successes and pitfalls, are documented in Steinhausen's work, it reflects a fascinating journey that has been inaccessible to the non-German speaking community. Therefore, the present compilation of translations, with accompanying introduction and discussion, was undertaken to allow a larger component of the vestibular scientific community to gain insight into peripheral labyrinthine mechanics provided by this historical account.

## Introduction

The early part of the twentieth century was a period of significant intellectual activity for vestibular physiology. It was known since the time of Valsalva (1) and Scarpa (2) that the labyrinth housed fluid-filled ducts that received branches of the *acoustic* nerve terminating in epithelia that were likely the origins of sensation, though the prevailing notion at the time was that they served auditory function. The presence of *hearing hairs* (*Hörhaare*) within the semicircular canal ampullae were suggested by Breuer as being important for signaling the direction and intensity of head movements (3). Confirmation that these projections from the surface of the crista were associated with hair cells was made by Retzius (4), whose exquisite drawings illustrated vestibular epithelia with “hairs” (stereocilia) that projected from the hair cells into the overlying cupula. The work of several investigators attempted to establish the direct correlate between semicircular canal function and head rotation-induced eye movements or postural reactions (3, 5–12), mostly following blockage of semicircular canals, selective lesions of the supplying peripheral branches of the VIIIth nerve, or eventually electric/galvanic activation of the sensory epithelia and corresponding nerve branches. This led to the meticulous dissections performed in the 1920s by McNally through which he and Tait deciphered the contributions of the different vestibular epithelia to balance and posture (13–15). Shortly afterward the first electrophysiologic recordings from vestibular afferents were conducted which laid the foundation for our current understanding of head movement encoding by vestibular afferent neurons through trains of action potentials transmitted to the central nervous system (16, 17).

The association between rotation-induced eye movements and sensory activation originating within the fluid-filled semicircular canals fostered questions concerning the kinematic relationship between the fluid within the canals and the sensory structures within the ampullae. These questions captured the interests of Breuer (3), Crum Brown (7), and Mach (10), who independently theorized that while the semicircular canals would move with the head during rotation, the inertia of the fluid within the canals would result in a force exerted by the endolymph within the semicircular canals. This became known as the *Mach-Breuer-Brown* theory of semicircular canal function. Though the theory was associated with their three names, only Breuer and Crum Brown advocated that head movements induce an endolymph displacement (3, 7). In agreement with the hypothesis advanced by Goltz (9), Mach proposed that fluid movement did not occur but that the pressure induced by the head rotation was sensed by the epithelia within the semicircular canal ampullae (10, 18). Mach's position was later taken up and supported by Breuer [see (19)].

Steinhausen endeavored to test the theory of endolymph movement through experiments in which movements of the cupula overlying the *crista ampullares* could be directly observed by visual inspection. He posited that the cupula would be displaced if head rotations resulted in endolymph movement. His original observations were made on a euthanized pike (20) using an approach which was subsequently further refined to enable observations from a more carefully and rapidly “freshly-produced” preparation (21). Thus, the main goal of the '31 paper was the demonstration that head rotation causes a movement of the cupula, ostensibly refuting the concept advanced by Mach (10) that the inertia of endolymph resulting from angular head movements imparted pressure upon the cupula without endolymph displacement. To ultimately address a principal criticism of his work arguing that his results were impacted by the isolation procedure and *in vitro* nature of the preparation, particularly purported by Wittmaack (22), Steinhausen repeated his experiments in a live preparation (23). Moreover, a major goal of this third investigation was to establish the relationship between cupula movements and eye movements, thereby confirming the fact that cupula motion generates excitation within the semicircular canal cristae during endolymph flow. This behavioral outcome further supported Steinhausen's claim that relative endolymph flow and cupular displacement was the foundation of semicircular canal function, and not due to a pathologic condition resulting from anomalies of the experimental preparation. In the '33 report, Steinhausen also differentiated between “normal,” short stimuli, which elicited tonic eye deviations and “abnormal” long-lasting rotations that caused a nystagmus. Collectively, the results from these experiments were quantified by a mathematical model of harmonic motion to describe cupular displacement in response to “step” stimuli (i.e., either at the onset or cessation of constant velocity rotations of different durations). This became the foundation of the analytical model of stimulus-evoked cupular displacement and referred to as the classic torsion-pendulum model (24, 25), driving investigations of peripheral vestibular physiology for decades to come [e.g., (26–28)].

The torsion-pendulum model provided a formal framework within which various attributes of semicircular canal physiology have been analyzed. It has been important not only for integrating the critical physical parameters of the semicircular canals and cupula that contribute to the encoding of rotational head movements, but also for identifying deviations from the model that reflect the contributions of non-mechanical characteristics of the coding cascade. Though Steinhausen's publications have been among the most oft-cited papers in the history of modern vestibular physiology, non-German readers have been deprived of a direct reading of this seminal work owing to the absence of a translation. This situation is remedied through this monograph. Translations of the 1927 (20), 1931 (21), and the “classical” 1933 Steinhausen (23) monographs are included, and are intended to provide practitioners of the field with the entire story of this classic experimental work. A summary follows the three translations in which Steinhausen's findings are briefly discussed in the context of contemporary knowledge of models of semicircular canal function. Our intention was not to provide a comprehensive review, but simply to provide some context regarding the importance of Steinhausen's findings to the conceptual questions of his day and their place in our understanding of semicircular canal function.

The original Steinhausen papers were translated from German into English by a native German speaker (HS) and subsequently edited by two native English speakers (MGP and LFH). The sentence-by-sentence translations reflect compromises between retention of the original style and sentiment of scientific publications from the early 1930s while making them linguistically accessible for present-day readers. This balance was challenging at times, particularly in view of the often long and tedious original German sentences and the very careful and often repetitious writing style of Steinhausen by which he attempted to be as precise as possible when referring to results and theories in the scientific literature. Nonetheless, all anecdotal advises and descriptions were maintained, but were occasionally supplemented by a note from the translator or editors. All original figures were reused and the labeling was translated and replaced to meet the quality requirements of contemporary publications. The error in the labeling in Figure 1 of Steinhausen's 1931 monograph (21), where the *ampulla* and the *barberry spine* in the original publication were reciprocally mislabeled, was maintained but explicitly noted in the translation.
