# Supplementary material for: Translations of Steinhausen's Publications Provide Insight Into Their Contributions to Peripheral Vestibular Neuroscience
Source: Front Neurol. 2021 Jun 4;12:676723. doi: 10.3389/fneur.2021.676723 (PMC8212934; doi:10.3389/fneur.2021.676723)
Supplement: Related texts

# Review article: On Visualization and Functional Inspection of the Cupula Terminalis in the Semicircular Canal Ampullae of the Labyrinth^1^

**Authors:** Wilhelm Steinhausen

**Affiliations:** Institut für Animalische Physiologie Theodor Stern-Haus, Frankfurt (Received June 1, 1927)

Over time, a number of theories about the excitation process in the vestibular organ have been put forward. A decision about which of these theories is the most correct could not be made until now, because the experimental foundations are not yet sufficiently known. A contribution to further knowledge of these experimental foundations will be given below.

## Historical Preface

The finer anatomical structure of the nervous endorgans in the semicircular canals of the labyrinth has been the topic of many studies. As early as 1858, *M. Schultze*^2^ described the hairs that rest on the crista ampullaris and protrude far into the semicircular canal lumen of rays and sharks, which as an “infinitely delicate, finely striated mass” form the cupula terminalis according to *Lang*.^3^ *M. Schultze*^4^ claims to have observed the cupula in isolation in the pike. As far as I could determine, a repetition of this observation including simultaneous functional testing has not yet been made.

The presence of hairs in the cupula terminalis was confirmed by all follow-up studies. Whether the agglutinative substance visible in the fixed preparation is an artificial product or not has become the subject of a lively dispute. *Hensen*^5^ has strongly advocated the opinion that only the hairs exist in life and the agglutinative substance is produced by the fixation. This conclusion is based on his observations of young, transparent fish (Gobius amongst others) in which only the hairs are visible, but not the agglutinative substance. More recently, all investigations have been performed almost exclusively on fixed preparations.

Any experimental data on the elastic properties of the cupula and its function during labyrinth stimulation is so far missing. There are only theories about it. Apart from the old *Mach-Breuer*^6^ theory of cupula movement, according to which the bending of the cupular hairs from their normal position by the endolymph flow should represent the physiological stimulus, a whole series of other theories have been put forward.^7^ Recently, the main question is how to reconcile the mathematical^8^ and experimental^9^ results about the short duration of the endolymph flow during adequate stimulation with the observations of the potentially very long-lasting stimulation symptoms (nystagmus, etc.). Of recent theories, special mention should be made in this context: the attempts of *Rohrer*^10^ to assume elastic after-vibrations of the cupula as the cause for the long post-duration symptoms and the theory of *Schmaltz*,^11^ according to which the cupula remains stationary during adequate stimulation and only a diffusion gradient at the cupula boundary is propagated by the endolymph flow. The resulting disturbance of a concentration equilibrium supposedly is the cause of the excitation.

Thus, our knowledge about the anatomical and physiological properties of the cupula terminalis is not yet sufficient to form a reasonably accurate picture of its effectiveness during stimulation of the labyrinth.

## Experiments on Vital Staining of the Cupula

All problems of the theories about the function of the semicircular canal organ could be resolved if it were possible to directly observe the cupula during labyrinth stimulation.

Of the various possibilities that would lead to a direct observation of the cupula terminalis, I first tried to use the method of vital staining of the cupula.

In frogs, one ampulla—usually the horizontal—was exposed.^12^ Then the whole preparation was submerged in a methylene blue-Ringer solution. After a certain period of time, the duration of which was subject to exploration, the preparation was returned to a dye-free Ringer solution.

In several cases, it was possible to visualize the unstained cupula through the reappearing translucence of the ampullary walls and also to demonstrate deflections during the endolymph flow. The flow in the semicircular canal was generated by compressing the semicircular canal with a glass rod, which, bolted to an arm of a micromanipulator, was pushed onto the semicircular canal.^13^ However, the staining succeeded only in so few cases that the method had to be abandoned in view of the difficulty of producing the preparation.^14^

## Direct Observation on Small Transparent Animals

In the literature, occasional statements^15^ can be found that in certain animals (Gobius species, perch, amphibians, larvae, etc.) the cupula or its hairs can be seen in the living animal from the outside without any dissection. I have not yet been able to study the respective species. In other small transparent and live fish, such as eel and grayling fry,^16^ small freshwater fish, including ornamental fish, I have often clearly seen the hairs at their base on the crista, with occasional indications of longitudinal stripes inside the ampulla. However, the cupula as a whole was never visible to such an extent that physiological experiments (rotation on the turntable or other means of flow generation in the semicircular canals) could have been performed.

## Incision of a Window Into the Ampulla

The method, which finally led to the intended success consisted of cutting a window into the wall of the ampulla, thus making the cupula accessible to direct observation.

## Methodological Details

The experiments were carried out on pikes, usually weighing 0.5–1 kg that arrived alive for the dissection. The right side of the skull was ablated and the brain was carefully removed. This provides a view from the inside onto the labyrinth, located freely in the cranial cavity, with a specific view of the utricle and the anterior vertical ampulla. The ampulla is exposed as far as possible. During this process, the ampulla as well as the entire labyrinth must not be touched. After this exposure, the cupula cannot be made visible by any means. I therefore believe that the claims made in the literature that it is possible to see the cupula inside the ampulla does not apply to freshly-produced preparations. Only when the cupula turns opaque due to phenomena related to the degeneration process it becomes visible from the outside with suitable illumination (strong transmitted light or lateral slit lamp illumination).

The opening of the ampulla requires a particular technique since the tissue of the ampulla is exceptionally flexible. Even with the micromanipulator, I have not been able to easily incise the ampullary wall, as it evades even the finest instruments. It is therefore necessary to harden the ampullary wall prior to the dissection, which can be easily accomplished without bringing more than the target piece of tissue in contact with the fixative.

As fixation solution, I used the following mixture reported by Nakamura^17^:

Müller's solution………243
4% Formalin solution………180
Acetic acid………16
Distilled water………540

The preparation, which is placed in 1.3x Ringer solution^18^ is slightly lifted out of the solution such that the ampullary wall emerges just above the surface. A trace of the fixation solution is applied to the ampullary wall with a fine rod, thereby hardening the wall. After some time, the preparation is completely re-submersed in the Ringer solution. At this point, the dilution of the fixation solution becomes so strong that no detrimental effect is induced in the rest of the tissue. With some practice, it is then possible to incise a window into the ampulla by free hand^19^ under the binocular loupe without damaging or even rupturing the cupula. As tools for cutting the window, I used tweezers, sharpened under the magnifying lens and splitters, made of Gillette blades, soldered to holders and sharpened at the tip—again under the magnifying lens. For illumination, a specially designed slit lamp from the Leitz company^20^ was at my disposal, with a 250-W high notch lamp replacing their illumination lamp. This type of illumination has considerable advantages for the dissection and was also used for the microphotography of the preparations.

Once the window has been cut into the ampullary wall, it is possible to see into the ampulla. Since transmitted light cannot be used, only lateral slit lamp illumination is possible. But even now the cupula is not immediately recognizable. The cupula is so transparent that its existence can only be inferred indirectly. In fact, with sufficient attention, debris can be detected, which accidentally has become trapped by the surface of the cupula, and which, in contrast to particles floating around freely in the fluid, occupy a relatively fixed position in space and thus render the outline of the cupula clearly visible. The fixed position in space is relative in the sense that fluid movements cause considerable movements of the cupula, which can be deduced from the motion of the adherent debris. Thereby, the cupula repeatedly returns to a defined resting position, which corresponds to the one that is theoretically to be expected. However, from this resting position the cupula can be very easily deflected. Particularly extensive movements can be observed by generating a flow within the semicircular canal through compression of the semicircular canal wall or by causing water movements when drops are made to fall onto the free surface of the fluid (cf. later).

However, endolymph flow, as corresponding to adequate stimulation, also causes noticeable cupula movements. If the ampullary window is covered with a fragment of a coverslip and the preparation, including microscope and illumination equipment, is placed on a turntable, then movements of the cupula are observed which, as far as can be deduced from subjective observations, correspond to those which should be expected according to the old *Mach-Breuer* theory.

Whether and to what extent cupular movements can occur, as assumed by *Rohrer* (cf. above) to explain the long duration of the nystagmus, cannot be reliably deduced from subjective observations. Also, to determine the natural oscillation frequency, the attenuation, etc. of the cupula, it appears necessary to supplement the subjective observations by an objective registration. Since a micro-cinematographic device was not at my disposal until now,^21^ I only can report about photographic images acquired with a still camera for the time being.

The debris, randomly adhering to the cupula is not sufficiently distinguishable from the background to produce photograms suitable for reproduction, even when the lateral slit lamp illumination is optimally adjusted. The reproduction of a photogram of the cupula in this state shall therefore be refrained from. Better images are obtained by artificially enhancing the contrast, which was achieved by applying Chinese ink according to Prof. *Bethe's* suggestion.

Chinese ink powder was mixed with 1.3x Frog Ringer solution and a trace of this solution was applied to the cupula using a finely pulled pipette.

Figure 1A (Image number 30) shows a photogram of the cupula obtained according to the described method. Figure 1B (Image number 29) represents the same preparation, captured under the same conditions with the only difference that during the capture a fluid movement was generated by dropping isotonic solution onto the exposed surface of the fluid in which the preparation was placed.

**Figure 1:** **(A)** Ampulla of the anterior semicircular canal of the pike with incised window in which the cupula (*Cup*) can be seen. *R* = edges of the window. Freshly-produced preparation. The dark areas are Chinese ink particles. Lateral slit lamp illumination. Image captured on May 30, 1927 at 5.30. Beginning of the dissection at 2.10. Exposure time 9 min (to obtain the best possible depth effect, the exposure was set to 3 min, respectively, at 3 different depths). Objective Leitz 3. Ocular Leitz 1. Magnification 50x (Image number 30) **(B)**. Same preparation. Cupula in motion (cf. text). Captured at 5.00, otherwise as **(A)** (Image number 29).

The equipment used for this purpose can be seen in Figure 2. A microscope slide (*T*) is placed above the object (*O*) to prevent image distortion caused by the movement of water on the surface. *M* is the microscope, *Sp* the slit lamp.

**Figure 2:** Device for the generation of fluid flow. *O* = Object, i.e., preparation with opened ampulla; *T* = Slide as protection against image distortion (cf. text); *M* = microscope; *Sp* = slit lamp; *R* = reservoir and tube for applying drops onto the water surface.

From the comparison of the two images, it can be seen that the cupula obviously moves extensively during the water movement. Its contour, which is clearly visible in Figure 1A, forms an arc in Figure 1B, the length of which allows determining the extent of the cupula oscillation. An image of the cupula movement during rotations on the turntable could not be achieved so far due to technical reasons. After all, the cupula appears to be very mobile, which must be taken into account in theories about the function of the semicircular canal organ. Deeper insight into the trajectory of the movement and the physical properties of the cupula can, of course, only be provided by the cinematographic recording of the cupula movement.

The method described here can also be used to study the currently relevant question^22^ on alterations of the cupula caused by the impact of heterotonic solutions and fixatives, as shown by several photograms obtained from fixed preparations.

Figure 3 shows an image of the fixed preparation. The crista and the epithelial layer of the zona lunata (*Z*), expanding above in a crescent shape, shine through the ampullary wall. There is no indication of the zona lunata and the crista in the unfixed preparation (cf. Figure 1). Above the zona lunata, the window (*F*) in the ampullary wall is visible, and through the window, the cupula (*Cup*) residing on the crista can be seen. Fixation causes the contour of the cupula to emerge in a clarity never seen in the unfixed preparation. Apparently, shrinkage has also been induced by the fixative.

**Figure 3:** Cupula of a pike. Fixed preparation. Pike with a weight of about 0.5 kg. Dissected on May 14, 1927. Image captured on May 18, 1927. Lateral slit lamp illumination. Exposure time: 4 min. *Cup* = cupula; *A* = ampulla; *F* = window; *Z* = zona lunata; *Cr* = crista (Image number 10).

Figure 4A shows an image of the cupula of a larger pike (about 2.25 kg). The ampulla has been removed and the ampullary wall has been ablated to a large extent. Part of the zona lunata is also removed, such that the cupula becomes visible almost down to its base at the crista. Since the cupula was freely accessible from both sides, it was possible in this case to capture images using transmitted light.

**Figure 4:** **(A)** Cupula of a pike. Isolated ampulla of the horizontal semicircular canal. Fixed preparation. The ampullary wall is completely ablated except for vestiges of the zona lunata (*Z*). Pike with a weight of about 2.25 kg. Dissected on March 8, 1927, image captured on May 20, 1927. Illumination: transmitted light. Exposure time: 1 min. Objective: Leitz 3, Ocular: Leitz 8. Magnification 80x (Image number 16) **(B)**. Same preparation as in **(A)**. Cupula in motion [cf. text for **(B)**]. All other as in **(A)** (Image number 17).

That the cupula can still be mobile despite fixation is shown in Figure 4B. The setting is exactly the same as in Figure 4A. The only difference is that a flow has been created by applying drops to the surface of the fluid in which the preparation was placed, as described in more detail in the text for Figures 1B, 2. The cupula has become much more rigid due to fixation compared to the freshly-produced state, but at least it remains sufficiently mobile such that distinct movements occur in the described experimental setting, as well as during rotations on the turntable. For demonstration purposes, this mobility also of the fixed cupula is very convenient.

Finally, Figure 5 depicts a cupula from the posterior vertical ampulla of a pike with a weight of 0.5 kg. The preparation shows the effect of fixation and a somewhat rough mechanical treatment. After all, it appears in better condition than some of the cupula images obtained by conventional histological methods presented in publications on the histology of the labyrinth.

**Figure 5:** Cupula of a pike. From the posterior vertical ampulla. Pike with a weight of 0.5 kg. Fixed preparation. Dissected on May 20, 1927, image captured on May 24, 1927. Transmitted light. Exposure time 1 min. Objective L3, Ocular L3. Magnification 80x (Image number 18).

The last images (Figure 4A and following) were taken with transmitted light. Since even stronger light sources could have been used in this case, images could also have been captured at higher magnification and thus the histological details could have been more distinctly highlighted.

Since we were more concerned with the function than with the histology of the cupula, the histological questions were postponed for the time being. Also, further efforts are directed mainly toward expanding the method of functional testing of the cupula, described here, to other animals, including intact preparations, in order to extend it in such a way that the physical properties of the living cupula (oscillation period, attenuation, etc.) can be accurately determined.

## Summary

A method is provided that allows exposing the cupula within the ampulla of the semicircular canal and studying its physical properties in freshly-produced preparations. The method essentially consists of incising a window into the ampulla and observing the cupula through the window under slit lamp illumination.

When creating a flow in the endolymphatic space (falling drops onto the exposed surface of the fluid within which the preparation is placed, or by rotation on the turntable), the cupula is deflected.

A series of photomicrographs of the cupula under various experimental conditions serves to demonstrate the applicability of the method.

## Footnotes

^2^*M. Schultze*, Arch. f. Anat. u. Physiol. 1858, P. 330–381.

^3^*Lang*, Zeitschr. f. wiss. Zool. 17. 1863.

^4^*M. Schultze*, a. a. O., P. 351ff.

^5^*V. Hensen*, Arch. f. Anat. u. Physiol., Anat. Abt., 1878, P. 466–489; 1881. P. 405–418.

^6^*Mach-Breuer* 1873/74. Cf. Nagel, Handbuch der Physiologie Bd. III, P. 778. 1905.

^7^Cf. e.g., Handbuch der normalen und pathologischen Physiologie 11, 797ff., 985ff. 1925.

^8^*W. Gaede*, Arch. f. Ohren-, Nasen- u. Kehlkopfheilk. 110, 6–14. 1923.

^9^*G. Rossi*, Arch. di fisiol. 13, 335–343. 1915. *Maier* und *Lion*, Pflügers Arch. f. d. ges. Physiol. 187, 47. 1921.

^10^*F. Rohrer*, Schweiz. med. Wochenschr. 1922, Nr. 27; cf. also *Rohrer* and *Masuda*, Handbuch der normalen und pathologischen Physiologie 11. 1925.

^11^G. Schmaltz, Pflügers Arch. f. d. ges. Physiol. 207, 127.

^12^With sufficient caution, this can also be done in the living animal.

^13^I reported on these experiments in a meeting of the med.-biol. evening at the University of Frankfurt on February 23, 1925, after I had first demonstrated the deflection of the cupula in our institute. Cf. Klin. Wochenschr. 4, 853. 1925.

^14^In retrospect, an explanation for the rather infrequent success of the staining experiments is tentatively explainable. The cupula may be potentially altered postmortem (coagulation?) to become stained by the used Methylene Blue-Ringer solution. If the solution exerts its effect on the cupula in a freshly-produced preparation, the cupula potentially becomes damaged and destroyed by the solution. I come to this conclusion because during the first attempts I was able to stain the cupula, however, later, when I had managed to work faster, I was not able to find the cupula at all, despite the most diligent efforts.

^15^*El. Fr. Schulze*, Arch. f. Anat. u. Physiol. 1862. Hensen, a. a. O. *G. Retzius*, Das Gehörorgan der Wirbeltiere Bd. II, P. 363.

^16^I am grateful to the authorities of the German Fisheries Association in Berlin and the Munich State Fish Hatcheries for providing me with fish stocks.

^17^*Y. Nakamura*, Beitrag zur Anatomie des Ohres 8, H. 1–3. 1914; cit. in *N. Satoh*, Der histologische Bau der Vogelschnecke. Basel 1917.

^18^1.3x Frog Ringer solution was isotonic for the pikes that I examined.

^19^The use of the micromanipulator, as I have found, is not advantageous at this point. The surgery only takes much longer because of the inconvenient setting.

^20^I would also like to take this opportunity to thank the Leitz company in Wetzlar for providing the lamp.

^21^But we hope to be able to make cinematographic recordings of the cupula movement at a later stage.

^22^Cf. *W. Kolmer*, Arch. f. Ohrenheilk. 116, 10–26. 1926 and *Wittmaack*, ibid p. 27–30.

# Review article: On the Evidence of the Movement of the Cupula in the Intact Semicircular Canal Ampulla of the Labyrinth During Natural Rotational and Caloric Stimulation

**Authors:** Wilhelm Steinhausen

**Affiliations:** Physiological Institute of the University Greifswald (Received April 25, 1931)

Some time ago, I described in this archive^1^ a method that allows visualizing the cupula in the semicircular canal ampullae of the labyrinth.

The method consists essentially in the fact that a window is cut into the exposed membranous ampulla and the cupula is observed through this window (*Note by the translators: even though Steinhausen used the word “window,” it is more likely a ruptured or pierced opening*). The shape and position of the cupula, which itself is completely transparent, can be deduced from the observation of the finest debris (ink or aluminum particles, or tissue debris, randomly floating in the preparation fluid, which adhere to the surface of the cupula).

As far as the physiological behavior of the cupula is concerned, the very large mechanical mobility of the cupula could be demonstrated, such that the original theory of the cupula function by *Mach-Breuer-Brown* was considered experimentally proven.

*Wittmaack*^2^ expressed concerns about my experiments. Based on his theory of the turgescence of the cupula, *Wittmaack* believes that by unintentional injuries during my preparation method, the surface of the cupula was destroyed. Its turgescence was thereby decreased to such an amount that like a liquid-filled bladder, which slackens by piercing, it had collapsed as a result of the injury and had been transformed into a mass of fibers floating hither and thither. The great motility is thus only a consequence of the injury. Also, symptoms related to degradation, as *Wittmaack* stipulated, could have changed the structure of the cupula in the sense of softening the tissue.

Although I do not share the view of *Wittmaack*, it was nonetheless desirable to find a gentler and faster preparation method. Also, for another reason, which seemed to me even more important, I aimed at an improvement of the method: the motility of the cupula in my earlier experiments was examined under conditions that were not quite physiological. The ampulla had been opened through the window and the flow produced in the surrounding liquid (either by movement of the liquid surface or by rotation) and affecting the cupula were of quite unpredictable magnitude and perhaps much stronger than those that occur in the intact ampulla during adequate rotational and caloric stimulation. It was therefore still possible to assume that during the small movements that occur during physiological stimulation in the endolymph, the cupula remains immobile. Thus, for the physiological stimulation, the various hypotheses that have replaced the deflection hypothesis may still apply, that is, the pressure hypothesis of *Wittmaack* among others, or the hypothesis of the movement of a diffusion gradient after *Schmaltz* among others.

It was therefore strongly desired to find a method that allows the cupula to be observed in the *intact* ampulla during adequate rotational and caloric stimulation and to reveal its mechanical behavior. In the meantime, I have developed such a method. In the process, I have found that the cupula is indeed also deflected under physiological stimulus conditions. It shows the deflections that are predicted by the movement of the endolymph according to the *Mach-Breuer* theory.

## Method

The experiments were preferentially carried out on the labyrinth of the pike, specifically on a freshly-produced preparation. The ampulla of the left anterior semicircular canal was exposed as described in the first publication (*Note by the translators: Steinhausen, 1927*). The preparation was then adjusted such that the semicircular canal was oriented vertically and the end of the semicircular canal ampulla (*Note by the translators: “end” refers to the region where the ampulla transitions into the semicircular canal*) emerged just slightly from the fluid (1.4-fold Ringer) in which the preparation is made. Thereafter a hole of about 0.1 mm in diameter was burnt into the wall of the semicircular canal using a pointed thermo cauterizer made of 0.1 mm thick platinum wire.

Care has to be taken that the semicircular canal does not extend too far out of the fluid, otherwise the wall will be slightly scorched by the heat of the thermo cauterizer. When adjusting to the correct height, the only effect at the contact with the thermo-cauterizer is a circular hole of about 0.1 mm in diameter.

Thereafter Ringer solution is added, so that the semicircular canal is completely submerged. Using a micromanipulator, a small funnel, with a diameter of about 0.1 mm at its lower end is placed above the hole, such that the end of the funnel reaches into the hole. Into the funnel, a trace of ink solution (some Chinese ink dissolved in 1.4x Ringer's solution) is added.

When applying the ink to the funnel, extreme care must be taken to ensure that fluid is not sucked out of the funnel by capillary phenomena. If one takes e.g., an ordinary glass rod to transfer the ink, and brings it to the funnel, fluid (*Note by the translators: endolymph from the semicircular canal*) will be sucked onto the glass rod. This leads to a strong flow within the semicircular canal and the ampulla and the cupula becomes detached. Such and similar incidents caused the loss of many preparations.

The ink solution is strongly cooled prior to the application. As a result of the cooling and its higher relative density compared to the endolymphatic fluid, it falls as a fine stream into the semicircular canal and the ampulla, attaches to the surface of the cupula and stains the cupula.

Appropriate positioning of the semicircular canal allows the most important parts for observation of the cupula to be stained. It is very important to avoid introducing too much ink into the ampulla; even more, one must prevent any debris from falling into the ampulla because even the smallest contaminant or the slightest agglomeration of ink particles can prevent movements of the cupula if these obstacles fall between the cupula and the ampullary wall.

As our experiments show, the cupula in a freshly-produced state has a size that causes it to almost touch the roof of the ampulla. There is only so little space between the cupula and the ampullary wall that a movement is just possible. The smallest particle of debris that gets stuck in this space fixes the cupula at the roof of the ampulla, making it consequently impossible to conduct physiological investigations on the movement of the cupula.

If the staining was successful, the hole in the ampullary wall is preferentially closed with a small spine, inserted into the hole. For closure, I use the most anterior tip of a barberry spine.

The described preparation method appears to me as the gentlest one possible, because the ampulla itself is not injured at all, rather a small hole is burnt into the end of the semicircular canal ampulla (*Note by the translators: “end” refers to the region where the ampulla transitions into the semicircular canal; see location of the barberry spine in* *Figure 1*). One might think that this manipulation could create a strong flow in the endolymph of the semicircular canal, and consequently injure the cupula. The experiment shows that this is not the case. Because the warming occurs at the highest place of the semicircular canal, and there is thus no reason that a fluid flow is generated. Thereby, the cupula remains completely at rest. The burning of the hole is a matter of moments, it suffices to simply touch the wall of the semicircular canal with the thermo cauterizer. It is evident that this is considerably time-saving compared with the previous method of cutting a window.

**Figure 1:** Stereo image of an ink-stained cupula in the intact semicircular canal ampulla of a freshly-produced pike labyrinth *[Note by the translators: the two labels indicated by* ^*^(i.e., Ampulla^*^and Barberry spine^*^) were erroneously placed in the original paper and should be swapped].

In addition, closing the hole with the tip of a spine is such a simple and gentle method, that any sort of noteworthy deflection of the cupula or even injury cannot occur.

This only leaves symptoms related to degradation as cause for potential changes of the physical state of the cupula prior to the observation of its physical behavior. In order to delay the occurrence of symptoms related to degradation as long as possible, we have always kept the preparation in rather chilled preparation fluid (*Note by the translators: 1.4x Ringer*). The time required for the preparation procedure from sacrificing the animal to studying the cupula movement under the microscope was about 1–1^1/2^ h. Explicit symptoms related to degradation under these conditions, however, occur only after many hours, sometimes only on the next day in such preparations. Therefore, I believe that the studies on the cupula movement can be considered to be made on a freshly-produced cupula.

After establishing the preparation method as indicated, it naturally is our next aim to investigate the cupula movement in living animals and I hope to be able to report on the respective outcome in the near future.

## Results

### Shape of the Cupula and Its Size Relation to the Ampulla

Figure 1 shows a stereo image of the stained cupula. The cupula is stained from both sides for greater clarity.

Figure 2 shows a cupula at higher magnification. The magnification is about 30x.

**Figure 2:** Ink-stained freshly-produced cupula in the intact semicircular canal ampulla of the labyrinth of a pike. Magnification about 25x.

As can be deduced from the figures, and as we have already concluded from our previous experiments, the hitherto existing assumptions—which could only rely on histological images of fixed preparations—about the size of the cupula in the freshly-produced state, are all more or less incorrect. The cupula is much larger than one has assumed until now, it almost touches the roof of the ampulla with its upper end. Also, its shape is much more complex than can deduced from the histological images.

In a forthcoming work, Mr. cand. med. *Fr. K. Wünn*, who kindly provided support during the preparation and the acquisition of photographic images of the cupula, will report on the shape of the cupula in the freshly-produced state and the influence on the shape by the fixative.

## Rotatory Stimulation

The produced preparation was placed on a turntable with the semicircular canal positioned horizontally and was observed under the microscope (Busch objective AO and eyepiece 5–15-fold). The microscope was adjusted such that the optical axis passed through the axis of rotation. When the turntable was set in rotation, the expected cupula deflections were seen most clearly. A mistake, such as by parallax errors, was completely excluded. However, in order to be able to exclude all objections, cinematographic recordings were made.

The cinematic apparatus (Zeiss-Kinamo) was mounted on the turntable and images were taken during the rotation.

The recorded movie will be presented at the upcoming Physiology conference in Bonn (Pentecost 1931).^3^

## Caloric Stimulation

More impressive is the deflection during caloric stimulation. The preparation was placed such that the semicircular canal was oriented vertically. At the lowest possible point of the semicircular canal, an electrically heated platinum loop (0.1 mm wire diameter) was placed. If current was passed through the platinum wire and as a result the platinum wire was heated, a deflection of the cupula, corresponding to the respective endolymph flow, became clearly visible under the microscope.

The deflection lasted, in contrast to the rotatory stimulation, very long, and after switching off the current, the cupula did not return immediately, but on the contrary the deflection usually increased further, and only after some time the cupula slowly returned to its initial position. The return movement lasted from many seconds to several minutes, depending on the magnitude and duration of the applied heat.

Figures 3, 4 show two images, which demonstrate the success of the caloric stimulation, i.e., the deflection of the cupula. Figure 3 shows a photographic image of the cupula at rest. The blackened edges indicate the prismatic shape of the cupula, which extends almost vertically above the crista in the intact ampulla. In Figure 4, an endolymph flow is generated by heating and the cupula is photographed on a stationary plate in three different positions. Figure 4 provides, as I hope, clear evidence for the large displacements of the cupula that can occur during caloric stimulation.

**Figure 3:** Ink-stained cupula in the intact semicircular canal ampulla of the labyrinth of a pike (freshly-produced) *at rest* (the 4 edges of the cupula are particularly clearly stained).

**Figure 4:** Same preparation as in Figure 3. *Caloric stimulation*. Three different deflection positions are photographically superimposed on a stationary plate (see also text).

Cinematographic images were also made of the deflections caused by caloric stimulation, which will also be shown at the Physiology conference in Bonn.^4^

## Conclusion

From the described experiments I believe I should see that the theory of *Mach-Breuer-Brown* on the function of the cupula has finally been proven experimentally. The observed deflections are in perfect agreement with calculations made by Mr. *Schmaltz* and the results about which he recently informed me.

## Summary

An improvement of the earlier reported method for visualizing the cupula in the semicircular canal ampullae of the labyrinth is described, which allows observations of the cupula in the *intact* semicircular canal ampulla and to study its behavior during physiological rotatory and caloric stimulation. This allowed producing a decision between the different theories on the function of the cupula.

I am very grateful for the loan of a micromanipulator, a preparation microscope and a stereo camera by the *Notgemeinschaft Deutscher Wissenschaften* and for the donation of a ball-bearing turntable by the *Vereinigte Kugellagerwerke* Fichtel & Sachs, Berlin-Schweinfurt.

## Footnotes

^1^*W. Steinhausen*, Pflügers Arch. 217, 747–755 (1927). Vgl. Z. Zellforschg 7, 513–518 (1928). Fol. otolaryng. 17, 410–415 (1929).

^2^*K. Wittmaack*, Über den Tonus der Sinnesendzellen des Innenohres, III. Arch. Ohrenheilk. 120, 256–296, spez. 270 (1929).

^3^Note edit in proof: has happened in the meantime.

^4^Note edit in proof: has happened in the meantime.

# Review article: On the Observation of the Cupula in the Semicircular Canal Ampullae of the Labyrinth in the Living Pike

**Authors:** Wilhelm Steinhausen

**Affiliations:** Physiological Institute of the University Greifswald (Received February 23, 1933)

After the method for dissection and visualization of the cupula had been established in freshly-produced preparations,^1^ it could, at least in principle, not be difficult to prepare the cupula also in the *living animal*. In the following, the method for preparing the cupula in the living animal^2^ and the results obtained with it shall be described. Subsequently, a theory of the cupula movement, which can be established based on the observations, shall be presented. All observations are made on the cupula of the left horizontal semicircular canal ampulla in the living pike, and the theory also applies only to the processes in this semicircular canal. It is very probable that for the other semicircular canals, certain differences in these processes with respect to those in the horizontal semicircular canal will be found. These differences, however, will likely not be of fundamental nature.

## Method of Preparing the Cupula in the Living Animal

As experimental animals, pikes were used, which originated from the Greifswald Bodden (*Note by the translators: shallow basin in the southwestern Baltic Sea, off the shore of Greifswald*) and therefore were adapted to saline water. The body weight of the animals was 750–1,000 g. For the preparation, an absolutely necessary requirement was a strict mechanical fixation of the skull while preserving the viability of the whole animal. This is achieved as follows: the animal is placed in a C-shaped *fish cradle* (*Note by the translators: custom-made mechanical fixation device to restrain the fish body for experimental manipulations*), similar to the construction used by *Tschermak*.^3^ Instead of movable arms of the *fish cradle* used by *Tschermak*, fixed bars are attached to a base rail. The bars are interconnected with wire mesh, which is covered with felt and cotton wool. This causes the fish body to be tightly wrapped by the padding except for the head and anus region. The opening on the top is secured by wide rubber bands, such that the animal is unable to make any movements, while on the other hand the bondage does not cause a compression: the head is clamped to a bite board and the skull is fixed laterally by 2 additional clamps. Bite board and clamps are firmly connected to the base rail of the fish cradle. This ensures a complete mechanical immobilization of the skull, without much struggling of the animal by the fixation. The cradle with the animal is placed in a container with isotonic Ringer's solution (1.4-fold Frog Ringer), aerated with oxygen. The animal breathes itself and remains completely calm.

Also, during surgery, no strong arousal occurs, provided the intervention progresses slowly. The *left horizontal ampulla* and a larger part of the corresponding semicircular canal are exposed. With some practice, the surgery is successful without collateral injuries and major bleeding. Once the ampulla and semicircular canal have been sufficiently exposed, one can either burn a hole into the semicircular canal with the thermo cauterizer, as described earlier on a freshly-produced specimen,^4^ or cut through the semicircular canal with a Gillette splinter knife. I lately came to prefer the latter method because it produces smooth cut surfaces and facilitates the insertion of cannulas (see below). When cutting through the semicircular canal, I put a very small piece of rubber underneath. When cutting, of course a strong ampullary stimulation occurs, which however subsides shortly after.

For *staining*, a small, ink-filled cannula is inserted into the semicircular canal. By applying pressure to a tube, connected to the cannula, ink can be introduced into the semicircular canal. After ink has been introduced into the semicircular canal, the cannula is withdrawn and the pike cradle is reoriented such that the ink can flow into the ampulla. Thereby the cupula is stained, precisely as described previously in freshly-produced specimen.^5^ If the staining is successful, the semicircular canal side of the cupula is made visible just as in the staining performed in the freshly-produced specimen; either as a sharp black line or more or less diffusely stained area.

Until the beginning of the staining procedure, *illumination* aspects pose no problem. During dissection and staining, very small optical magnifications are sufficient. Accordingly, the distance between object and lens is very large and illumination can be provided from above by strong microscopy lamps. When observing the cupula, in particular at higher magnifications, the illumination is insufficient and adequate illumination becomes a difficult task. This problem could be solved, however, if the newly developed top-light illumination methods (*Ultropak-Leitz*, episcopic illumination—*Zeiss, Busch*, etc.) could be applied to the observation of the cupula. Unfortunately, that is not possible. This is due to the fact that all of these illumination methods are designed exclusively for surface observations. However, the cupula is located deep inside the tissue, in a tissue funnel. The opening of the funnel cannot be sufficiently enlarged such that the wide lens sockets of the illumination devices in question can be positioned sufficiently close. Until the size of the sockets of the top-light illumination devices can be made smaller, any application in physiology remains limited.

One might think of generating a picture of the cupula through a suitable photographic lens, which is then observed microscopically.^6^ My own experiments have shown that this method likely allows a better observation, which is why this method should be tested further.

Good observation of the cupula in the living animal has so far only been achieved by providing illumination through the pharynx. A strong low-voltage lamp was inserted into the pharynx allowing observation by translucent light. For subjective observation, the achieved light intensity is entirely sufficient. Photographic and especially cinematographic recordings, such as those made during the observation of the cupula in the freshly-produced specimen,^7^ have not yet been successful in the living animal. This is due to the thick layer of tissue between the horizontal ampulla and the pharynx, which cannot be removed without damaging the labyrinth or harming the animal. Since this layer of tissue absorbs a very large amount of light, the brightness is insufficient for photographic image acquisition.

For the time being, it was thus necessary to limit the focus in the living animal to subjective observations. This however, suffices to clearly ask the essential questions. When creating a flow in the semicircular canal, it is possible to subjectively observe very well the deflection of the cupula and the reaction of the animal (eye movements etc.). As described earlier,^8^ one can observe the cupula and the eye simultaneously with the help of a drawing mirror. However, a simpler and better approach is the use of multiple observers; one observer for the behavior of the cupula and the other for e.g., the eye movements.

## Results

### Rotatory Stimulation

It would have been most desirable to apply the same stimulation methods also in the living animal as used to observe the cupula movement in the freshly-produced specimen.^9^ So, the most obvious procedure would have been to put the animal on a turntable and to observe how the cupula behaves and how the animal reacts, i.e., which eye movements, etc. occur. However, a simple consideration shows that this would not have been the most straightforward approach to obtain information about the more detailed mechanism of the semicircular canal excitation. First, the technical task of rotating the living animal together with the surrounding water tank and the rest of the device, while simultaneously observing eye and cupula movement in detail, would have been rather difficult to achieve given our limited resources. But even if we had succeeded in solving this task, the result would not have been unequivocal. In fact, rotation would not only cause the expected deflection of the cupula of the observed ampulla (in our case the left horizontal ampulla), but also rotational effects in other parts of the same labyrinth, in particular of the cupula in the horizontal ampulla of the other, right labyrinth. Eye movements observed during rotation (so far, we restricted our observations to eye movements) could therefore not be simply assigned to the movement of the cupula in the observed ampulla. It would thus well be possible that the observed eye movements would have been caused by rotational effects at the other, unobserved sites.

## Direct Mechanical Stimulation of the Left Horizontal Semicircular Canal

The currently best approach to make a particular statement about the detailed mechanism of the cupula activation rather derives from the direct, isolated stimulation of an ampulla with a concurrent observation of the cupula and the eyes. The method is indeed applicable because the rotational effect on the cupula has already been demonstrated by the observations on the isolated preparation. As shown in previous studies,^10^ rotation causes deflections of the cupula as predicted by the *Mach-Breuer-Brown* theory. The goal is now to investigate how cupula movements are linked with eye movements. So, it is sufficient to cause a cupula movement by the simplest means and to observe the corresponding eye movements.

Isolated stimulation of individual ampullae (of course without observation of the cupula) have been carried out quite frequently (*Flourens, Breuer, Ewald* and many others.^11^) A particularly elegant method of isolated stimulation of semicircular canals in the pigeon was applied by *Ewald*^12^ in his famous experiments using the pneumatic hammer.

Fishes have been used by *Bethe*,^13^ *Lee*,^14^ *Kubo*,^15^ *Maxwell*^16^ and others to conduct stimulation experiments. The results of all these experiments have not yet led to a consolidated opinion about the function of the semicircular canal system. For example, *Maxwell*^17^ writes about E*wald's* experiments that indeed the latter confirmed the fact of a linkage between an ampulla with movements of the eyes in a particular plane. Further implications of his experiments that endolymph movement in one direction causes excitation, and inhibition in the other are “open to criticism.” Many contradictions, contained in the literature on semicircular canal excitation can be resolved by observations of the cupula during isolated semicircular excitation. Since this has not been possible so far, it explains, in my view, the uncertainty in the interpretation of the hitherto existing experimental results.

To determine the dependency of eye movements on the movement of the cupula, the *left horizontal ampulla* is mechanically stimulated in isolation while the movement of the cupula and the eyes are observed.

The isolated mechanical stimulation of the cupula was performed as follows: a cannula is inserted into the semicircular canal.^18^ The cannula is connected to a tube system, which allows applying a shorter or longer lasting pressure pulse to the other end using an appropriate device. *Breuer*^19^ has already inserted cannulas into the semicircular canals but failed to obtain reliable results. *Bethe*^20^ has also made experiments with the insertion of cannulas into the semicircular canal, which, however, as he states, were unsuccessful.

If the cannula is positioned in the ampullary part of the semicircular canal, generation of pressure in the tube system should cause an endolymph movement in the semicircular canal directed toward the ampulla (i.e., ampullopetal flow.^21^) Observation of the cupula, reveals a deflection in the direction of the utricle. It is possible now to adjust the magnitude and timing of pressure application *such that a clearly visible*, but small *deflection of the cupula becomes visible*. Such a small, rapidly fading deflection of the cupula causes a *horizontal deviation of both eyes to the right*. The eyes remain in the new position and only return to their normal position after a while. If the deflection of the cupula is maintained for a longer period of time (continuous pressure exerted onto the tube connected to the semicircular canal cannula), the deviation changes into a *horizontal nystagmus of both eyes with the fast component directed to the left*.

During a *reverse deflection of the cupula*, i.e., toward the semicircular canal (ampullofugal flow), *there is no effect on the eye position*. The eyes remain in their normal resting position.

A dependency of semicircular canal excitation on the flow direction of the endolymph has already been found by *Ewald*^22^ in his experiments with the pneumatic hammer in pigeons. *Ewald* discovered that an ampullopetal endolymph flow caused a strong excitation, and an ampullofugal endolymph flow caused a weak excitation. He did of course not investigate the behavior of the cupula.

These observations indicate that in fact cupula movements cause the excitation of semicircular canals during endolymph flow rather than other changes of the state of the cupula (direct pressure effects, osmotic changes, ion shifts or the like).

## Electrical Stimulation

Flow in the left horizontal ampulla in ampullofugal direction, i.e., anode on the utricular side of the ampulla and cathode on the semicircular canal side, cause *deviation of both eyes to the right* (current threshold magnitude about 0.6 mA). Current flow in the opposite direction *fails to provoke any eye movements* (even not at magnitudes beyond 5 mA). During current stimulation, the *cupula remains completely at rest*. *Brüning's* theory^23^ on the cataphoretic effect of the electric current on the cupula thus fails to explain the facts.

Switching to faradaic stimulation by using a large sled inductor, while maintaining the same position of the electrodes, yields no semicircular canal excitation. Augmenting the current magnitude by increasing the overlap of the coils of the inductor, causes the impact of the current loops to more likely evoke a general excitation of the surrounding muscle tissue than to produce a distinct semicircular canal excitation. This suggests that the chronaxie of the semicircular canals must be unusually large. For the entirety of the vestibular apparatus, *Bourguignon*^24^ has already stated a particularly large chronaxie.

## General Conclusions From the Observations

Since the cupula, as demonstrated, remains immobile during electrical stimulation, the electrical stimulation of the semicircular canal system must result from a direct stimulation of the sensory epithelium or of the ampullary nerve itself. The following will therefore only discuss the processes during rotational stimulation. It has been shown that a movement of the cupula of the left horizontal ampulla toward the utricle causes a deviation of both eyes to the right or, during a prolonged deflection of the cupula, causes a nystagmus to the left. This result complies with the prediction of the *Mach-Breuer-Brown* theory of semicircular canal excitation.

Figure 1 schematically illustrates the relationship between the cupula of the left horizontal semicircular canal (l.h.s.c.) and eye movements.

**Figure 1:** Head of a pike viewed from dorsal with delineated left horizontal semicircular canal and utricle (schematic).

At the onset of a leftward rotation from a static position (large arrow), according to the theory by *Mach-Breuer-Brown*, the endolymph, due to its inertia will lag the motion of the semicircular canal wall. This will cause a relative motion of the endolymph in the direction indicated by the small arrow. Accordingly, an ampullopetal flow must be caused in the semicircular canal. This ampullopetal flow induces a deflection of the cupula^25^ in the direction of the utricle.

To avoid a shift of the point of fixation on the retina, the eyes will perform a rightward counter-rotation. Ampullopetal endolymph flow in the left horizontal semicircular canal and consequent deflection of the left horizontal cupula toward the utricle must therefore be associated with a rightward deviation of the eyes. Since an artificial deflection of the cupula toward the utricle causes a rightward eye deviation, it follows that the *cupula deflection during rotation represents the actual stimulus that triggers the eye deviation*.

In the same way, it can be demonstrated that during a *prolonged leftward rotation*, a long-lasting deflection of the left horizontal cupula toward the utricle must occur. During prolonged leftward rotation, a *nystagmus to the left* can be observed in the intact animal. Since prolonged artificial deflection of the cupula of the left horizontal ampulla provokes a nystagmus to the left, it can be concluded that the nystagmus to the left during prolonged rotation derives from a deflection of the cupula of the left horizontal ampulla during the ampullopetal endolymph flow toward the utricle.

Finally, the same line of arguments allows concluding that the *after-nystagmus to the left*, which is observed after a longer, rightward rotation in the intact animal, is triggered by a continuing endolymph motion, as must be deduced from the theory by *Mach-Breuer-Brown* and my own experimentally verified deflection of the cupula of the left horizontal ampulla toward the utricle (see also below).

## On the Theory of Cupula Movement

There thus is a clear dependency of the labyrinthine symptoms (eye movements) on the *deflections of the cupula*. The reported observations are thus definitive proof for the validity of the *Mach-Breuer-Brown* theory. In addition, the results of the experimental study allow formulating a theory of the cupula deflection, which will be described following the theoretical work of *Gaede, Rohrer*, and *Schmaltz*^26^ below.

Figure 2 shows schematic drawings of the semicircular canal, ampulla and cupula. B is the semicircular canal, E, the endolymph within the semicircular canal, A, the ampulla, and C, the mobile cupula, with the crista (Cr) as pivotal point. Because of the endolymph-tight closure, any endolymph movement must induce a cupula movement and *vice versa*. The endolymph is thus mechanically tightly coupled to the cupula^27^. This tight coupling can be accurately demonstrated in experiments. If the semicircular canal cannula (see above) is tightly inserted into the semicircular canal, the cupula will remain in any possible position, depending on the pressure, produced by the tubular system connected to the cannula. Only when the endolymph is given an evasion alternative e.g., by inserting the cannula only loosely into the semicircular canal, the cupula, due to its mechanical force, returns to the average resting position by shifting the endolymph.

**Figure 2:** Semicircular canal with ampulla and cupula (schematic).

Due to the mechanical force of the cupula, the system has a null position from which it can deviate by rotation in the plane of the semicircular canal. The mechanical force of the cupula causes a return to its null position. This theory thus leads to *harmonic equations*. The exact equation can be written as follows:

(1) $$\begin{array}{l} m\frac{d^{2}x}{dt^{2}}+k\left(\frac{dx}{dt}-\frac{d\xi }{dt}\right)=-a^{2}\left(x-\xi \right) \end{array}$$

In this equation, x is the motion coordinate of the cupula (or rather of the endolymph), ξ the motion coordinate of the wall, *t*, time, m, mass of the endolymph including the relatively small mass of the cupula, *k*, the friction coefficient and *a*^2^, the constant of the mechano-elastic force that causes the cupula to return to the null position. If *a*^2^ = 0, this is neglecting the influence of the mechano-elastic force of the cupula on the movement, then:

(2) $$\begin{array}{l} m\frac{d^{2}x}{dt^{2}}+k\left(\frac{dx}{dt}-\frac{d\xi }{dt}\right)=0 \end{array}$$

Equation (2) is the equation of pure endolymph movement, assumed by *Gaede*^28^ and which was at length discussed with respect to rotatory and caloric stimulation by *Schmaltz*.^29^

The equation for the cupula movement Equation (1) can be written as follows:

(3) $$\begin{array}{l} m\frac{d^{2}x}{dt^{2}}+k\frac{dx}{dt}=-a^{2}x+a^{2}\xi +k\frac{d\xi }{dt} \end{array}$$

This is the equation of a forced, damped oscillation in which the term $a^{2}\xi +k\frac{d\xi }{dt}$ can be interpreted as a disturbance function. In order to be able to solve the equation, first of all the disturbance function must be known. The simplest usable case for our purpose is obtained by setting $\frac{d\xi }{dt}$ = constant = *w*.^30^ In this case ξ = ∫ *wdt* = *wt*. This indicates that during the period in question the rotation velocity of the wall is constant. Thus, for the semicircular canal wall a sudden transition from rest into a movement with constant speed, or *vice versa*, is assumed, an assumption that can be practically met with sufficient approximation.

The equation for the cupula movement Equation (3) for this case is:

(4) $$\begin{array}{l} m\frac{d^{2}x}{dt^{2}}+k\frac{dx}{dt}=-a^{2}x+a^{2}wt+kw \end{array}$$

According to the experimental findings, *k* is very large and *a*^2^ is small. The general solution^31^ of Equation (4) therefore is:

(5) $$\begin{array}{l} x=\frac{u_{0}-w+x_{0}\beta_{2}}{\beta_{2}-\beta_{1}}•e^{-\beta_{1}t}-\frac{u_{0}-w+x_{0}\beta_{1}}{\beta_{2}-\beta_{1}}•e^{-\beta_{2}t}+w•t \end{array}$$

Therein, *x*_0_ is the cupula coordinate at the time *t* = 0, *u*_0_ the velocity of the cupula at time t = 0, β_1_ and β_2_ have the following values:

$$\begin{array}{l} \beta_{1}=\frac{k}{2m}+\sqrt{\frac{k^{2}}{4m^{2}}-\frac{a^{2}}{m}}\\ \beta_{2}=\frac{k}{2m}-\sqrt{\frac{k^{2}}{4m^{2}}-\frac{a^{2}}{m}} \end{array}$$

Since ξ (i.e., the motion constant of the semicircular canal wall) = *wt* (see above), it follows for the relative motion of the cupula (*z*) with respect to the wall z = x - ξ:

(6) $$\begin{array}{l} Z=\frac{u_{0}-w+x_{0}\beta_{2}}{\beta_{2}-\beta_{1}}•e^{-\beta_{1}t}-\frac{u_{0}-w+x_{0}\beta_{1}}{\beta_{2}-\beta_{1}}•e^{-\beta_{2}t} \end{array}$$

It is only necessary to insert into this equation the values β_1_, β_2_ and w and for the initial conditions x_0_ and u_0_ in order to obtain z, i.e., the cupula deflection as a function of the time *t*.

In the following, the *general character of the cupula movement* will be examined, in particular regarding the dependency on the rotation time or rather the turning angle. Assuming that the rotation is always performed at the same velocity, the rotation times are a direct indicator for the turning angle. It shall therefore be investigated how the cupula movement changes when the turning angle (or the rotation time) changes. In particular, the question is how the cupula movement during small turning angles, i.e., short rotations, differs from those during large turning angles, i.e., long-lasting rotations. Short rotations form in fact the normal way of operation of the vestibular apparatus, as occurs in daily life, while longer-lasting rotations occur only under exceptional circumstances. During short rotations, appropriate compensatory movements, for example the illustrated eye movements are triggered, while during abnormal, longer-lasting rotations a nystagmus and subjective dizziness is provoked. In the calculation, 20 s (=15 divisions in Figure 3) is assumed as the longest rotation time, completing 10 full turns. 1 scale mark therefore indicates 240° turn angle. Concerning the cupula movement, only relative numbers are calculated. From the above indicated prerequisites, the following result, illustrated in Figure 3, is obtained.

**Figure 3:** Cupula movement during long rotation periods. Abscissa: time (scale marks 30 = 40 seconds); ordinate, cupula movement as arbitrary scale.

In Figure 3, the abscissa represents time from 0 to 30 (corresponding to 40 s), the ordinate indicates the cupula deflections, plotted arbitrarily from 0 to 1. The assumption is made that the semicircular canal at time t = 0 is put into a uniform rotation at the speed u_0_. The solid curve indicates the cupula movement. As can be seen, the cupula very quickly reaches maximum deflection, which in the indicated case assumes a value of 0.85, and then returns very slowly to the resting position. At time 15, i.e., after 20 s, this resting position is essentially reached (theoretically the cupula returns to its resting position only after an infinite amount of time). At time 15, the movement is stopped, i.e., the rotation is suddenly terminated. The solid line indicates the movement of the cupula when stopping. This causes a rapid deflection of the cupula to the other side, with the same magnitude as during the rotation onset, followed by a slow return motion to the resting position, resulting in the same curve as following the onset of the rotation.

Long rotations cause in fact a long-lasting cupula deflection, which is followed by a similar deflection to the other side when the rotation is stopped. Long-lasting cupula deflections are associated with a nystagmus. By additionally considering the events in the corresponding semicircular canal on the other side, the curves demonstrate that a rotational nystagmus during turning is followed after stopping by an oppositely-directed nystagmus.

The curves also explain the long duration of the rotational nystagmus as well as that of the after-nystagmus. The plot also contains the curve representing the endolymph movement (dotted line), as can be deducted from *Gaede*'s theory, assuming i.e., that the mechanical force of the cupula is zero (see above). The endolymph movement is pulsatile, both at the time of rotation onset and at stopping, and thus by itself cannot explain the long duration of the after-effect. Only when considering the cupula, the long duration of the nystagmus and the after-nystagmus becomes explainable.

Figure 3 also shows the cupula movement under the conditions that the stopping occurs before the cupula has reached null position. For example, the curve τ = 6 indicates that at time 6 the rotation is interrupted and corresponding curves after stops at shorter periods are indicated.

For very short rotation periods, the curves would be densely overlapping, thus preventing an overview.

Therefore, Figure 4 illustrates separately the curves for cupula movements during short rotation periods. Accordingly, the curve τ = 0.1 indicates the cupula deflection calculated when the rotation was stopped after the time τ = 0.1, corresponding to a turning angle of 24°. Such small turning angles, only cause a very brief cupula deflection (a nystagmus can therefore not be triggered) and the post-rotational movement (as well as the after-nystagmus) becomes smaller and smaller and finally imperceptible. Small turning angles thus cause a very different form of cupula movement as large rotation angles: the cupula movement during small turning angles mainly consist of a short deflection with a magnitude that decreases with decreasing turning angles. The analysis thus explains the fact that during small rotations (representing normal semicircular canal stimuli) it is exclusively the magnitude of the rotation that is differentially perceived. Only during longer-lasting rotations (abnormal semicircular canal stimuli) the perception of the rotation is superimposed and impaired by the gradual return movement of the cupula (nystagmus) as well as by the oppositely-directed negative deflection of the cupula (after-nystagmus) after stopping of the rotation. The curves also show how the duration of the nystagmus and after-nystagmus increases with increasing rotation period and which quantitative relationship can be deduced. Experimentally, this dependency has been particularly well-studied by *Masuda*^32^ (see *Rohrer*).

**Figure 4:** Cupula movement during short rotation periods. Descriptions as in Figure 3.

The theory described here only explains the processes in the semicircular canals of the pike. It is well possible that in other animals including humans, adaptations might exist and influence the processes, such as for example different constants compared to those obtained by the experimental observations on the pike labyrinth.

## Summary

1. The method, described in earlier studies, also allows visualizing the *cupula* in the *living* pike by ink staining and to determine its mechanical behavior.
2. *Electrical stimulation* does not elicit cupula movements, although the typical labyrinthine symptoms are present, of which only eye movements were observed in the present study. *Brüning's* theory of the cataphoretic deflection of the cupula by electric current does not meet the facts. The electric current is effective only in utricle-semicircular canal direction, an inverse direction fails to yield an excitation *(Note by the translators: the asymmetric effect of current stimulation is likely related to the placement of one electrode close to the cupula and the other at a distance; reversal of the polarity would thus yield only a single effective configuration)*.
3. Isolated mechanical stimulation of the *left horizontal ampulla* in the living pike while observing the cupula was made as follows: a cannula, connected to a tubing system was inserted into the semicircular canal. Application of pressure to the tube caused a displacement of the endolymph in the semicircular canal. The result was the following:
  - The cupula is tightly coupled to the endolymph. Deflections of the cupula can only be reversed if the endolymph moves.
  - Deflection of the cupula toward the utricle causes a *movement of both eyes to the right* if the cupula deviation is brief, and a *nystagmus of both eyes to the left*, if the cupula deviation is longer-lasting. Deflection of the cupula toward the semicircular canal fails to yield an excitation.
4. Based on the experimental results, a theory of the cupula movement is established, which closely follows the work of *Gaede, Rohrer* and *Schmaltz* on the theory of endolymph movement.

I am very grateful to Dr. *Erdmann*, Medizinalpraktikant *Ulrich*, and Medizinalpraktikant Dr. *des. Wünn* for their help with the preparations. I also thank the *Notgemeinschaft der Deutschen Wissenschaft* for providing a micromanipulator and a dissecting microscope.

## Discussion

In his quest to resolve questions regarding how the semicircular canal cristae transform head motion, through the action of the endolymph, into neuronal signals and eventually into motor commands for eye movements, Steinhausen provided several key features of the mechanism:

1. he demonstrated that rotational head movements result in relative endolymph flow and a force exerted upon the semicircular canal cupula producing its displacement;
2. he demonstrated a tight coupling of the cupula with the endolymph;
3. he demonstrated that, in the pike, unilateral (i.e., *via* a cannula in the left horizontal semicircular canal) small cupula deflections in the ampullopetal direction (toward the utricle) caused compensatory movements of both eyes; oppositely directed (i.e., ampullofugal) unilateral cupula displacements did not produce detectable eye movements.

Collectively, these findings were interpreted to refute other hypotheses concerning endolymph and cupula function that prominently circulated at that time. This was clearly expressed in the summary of the '33 monograph (23): “*These observations indicate that in fact cupula movements cause the excitation of semicircular canals during endolymph flow rather than other changes of the state of the cupula (direct pressure effects, osmotic changes, ion shifts or the like).”* These observations represented hallmark features of the vestibular periphery, which were experimentally demonstrated for the first time (despite limitations concerning the exact mechanical behavior of the cupula, see below). Through these observations Steinhausen recognized the similarity to harmonic oscillations and advanced the quantitative relationship between the force induced by rotational head movements and the mechanical factors influencing cupula motion. In doing so, he conceptualized the sensory transformation of angular head movements. While it wasn't until more than a decade later that van Egmond et al. (25) applied the *torsion pendulum* label to this transformation, it is readily traced back to Steinhausen's seminal observations culminating in the '33 paper (23). This formulation had a tremendous impact on the field of peripheral vestibular physiology by ushering in the first quantitative model of peripheral vestibular function, representing the seed from which numerous quantitative descriptions have grown [reviewed in (29)]. Therefore, while Steinhausen did not originally refer to his model as a torsion-pendulum, he was the first to apply a rigorous mechanical analysis to semicircular canal function and proposed the essential form of the mathematical model that shortly thereafter came to be known by that metaphor (23).

Despite the significance of Steinhausen's biomechanical model of endolymph-cupular interaction (23), the experimental results and subsequent interpretations are associated with significant irony. The principal conclusions regarding endolymph movement and tight coupling with the cupula were based upon the readily-observed cupula displacements. These were documented in the photograph taken during caloric stimulation [Figure 4; in (21)] and the summary schematics illustrating the *swinging-gate* cupula motion profile [Figures 1, 2; in (23)]. However, more recent investigations demonstrated cupular displacements exhibiting much more modest profiles resembling a diaphragm, with its perimeter attached to the ampullary walls and coupled to laminar flow profiles of the endolymph exhibiting considerably smaller magnitudes (30, 31). This discrepancy may be explained by cupulae that became detached from the ampullary walls during Steinhausen's manipulations (e.g., creating the window to observe cupular motion, or during creation of the fenestra through which ink particles were introduced into the endolymph). The irony in Steinhausen's interpretations, then, rests in the strong likelihood that the observations were derived from the non-physiologic condition represented by detached cupulae.

Meanwhile, as a champion of the concept advocating the fundamental importance of interstitial hydrostatic pressure to physiologic function, Witmaack (22) challenged the veracity of Steinhausen's findings from the *in vitro* preparations. Witmaack argued that this turgescence would be lost in the *in vitro* condition, casting the analogy of the cupula to a balloon, and the flaccid condition resulting from the loss of turgor or surface rupture (22). Consequently, Steinhausen worked to improve his methods of preparation so that the observations were closer to the *in vivo* condition. Indeed, the observations from the living pike, being similar to those from the earlier *in vitro* preparations, argued against Witmaack's (22) challenge and supported Steinhausen's claim that large cupula displacements were not a consequence of the *in vitro* condition, *per se*. However, it appears highly likely that his interpretations were based upon observations of detached cupulae, independent of whether they were made under *in vivo* or *in vitro* conditions.

Despite the likelihood that Steinhausen's conclusions were derived from observations of detached (and, therefore, non-physiologic) cupulae, this condition resulted in an overall positive impact on the understanding of the biomechanical characteristics contributing to semicircular canal function. To be sure, cupula detachment would have a profound impact on these characteristics. For example, Rabbitt et al. (31) reported a dramatic reduction in the adaptation time constant in cupulae similarly detached as depicted by Steinhausen (21). However, the detached cupulae actually made it possible for him to observe the rotation-evoked endolymph displacement and coupling with the cupula, fostering the postulates regarding the application of harmonic oscillations and the mechanical factors contributing to endolymph displacement. Ironically, had the preparation been made to retain the normal *in situ* attachments, it is highly unlikely that Steinhausen would have observed the displacement of the central cupula that has been shown to be far less than 1 μm (31). Without these observations, Steinhausen would likely have conceded that sensory transduction was founded in other mechanisms under consideration at that time.

Of course, the alternative mechanism of which Steinhausen was eminently aware was advocated by Mach (10), whereby angular head movements generated pressures acting on the cupula, and implied that the cupula was an integral component of a pressure transducer. We now appreciate that these “competing” hypotheses (e.g., endolymph displacement and pressure generation) are not mutually exclusive, and that pressure differentials across the cupula lead to small displacements. For example, Oman and Young provided an analytical formulation of pressure generation resulting from angular head movements, which was consistent with human vestibular subjective thresholds (32). They also predicted that pressures acting on the cupula would result in diaphragm-like cupular displacements, profiles that were observed experimentallys shortly thereafter (30). The pressures leading to small cupular displacements (33), in the range of millipascals, have been shown to be sufficient to modulate the discharge in vestibular afferent neurons (34). Therefore, while Steinhausen's view of cupular movement was overzealous, the logic of the generative biomechanical model laid the foundation for decades of vestibular physiology research.

## Author Contributions

HS translated the original German text to English, contributed to the writing, and editing of the Introduction and Discussion. MGP edited the translations, Introduction, and Discussion. LFH contributed to the writing of the Introduction and Discussion and edited the translations. All authors contributed to the article and approved the submitted version.

## Conflict of Interest

The authors declare that the research was conducted in the absence of any commercial or financial relationships that could be construed as a potential conflict of interest.

## Footnotes

^1^*Steinhausen, W*.: Pflügers Arch. 217, 747–755 (1927); 228, 322–328 (1931); Z. Zellforsch. 7, 513–518; Z. Laryng. etc. 17, 410–415 (1928); Z. Hals- usw. Heilk. 29, 211–214 (1931).

^2^In Arch. Ohr. etc. Heilk. 132, 147 (1932) I have reported the successful visualization of the cupula in the living animal already in February 1932. If the detailed publication only takes place now, then external reasons (illness and other adversities that kept me from working) were decisive for the delay.

^3^*Tschermak, A. v*.: Pflügers Arch. 222, 439–444 (1929).

^4^See *W. Steinhausen*: Pfügers Arch. 228, 322–328 (1931).

^5^Occasionally, the staining has to be carried out several times, because as a result of the reverse cupula movement (see later), the amount of injected ink is washed out, causing pulsations that are in synchrony with the respiratory rhythm.

^6^Herr Prof. *Seeliger* indicated that for the microscopic observation of filaments, this procedure is very successful.

^7^See *W. Steinhausen*: ibid.

^8^Arch. Ohr. etc. Heilk. 132, 148 (1932).

^9^*Steinhausen*: See literature in footnote 1, previous page.

^10^*Steinhausen, W*.: ibid.

^11^With respect to literature, see e.g., *Bethes* Handbook Vol. 11 1, Rezeptionsorgane. 1926; and Vol. 15 1, Correlations 1930 and Vol. 18. Supplement 1932. In particular, the articles by *M. H. Fischer* und *Magnus und de Kleijn*.

^12^*Ewald, I. R*.: Physiologische Untersuchungen über das Endorgan des Nervus octavus. Wiesbaden 1892.

^13^*Bethe, A*.: Biol. Zbl. 14, 97–114, 563–582 (1894).

^14^*Lee, F. S*.: J. of Physiol. 15, 311–348; 17, 192–210 (1894).

^15^*Kubo, I*.: Plügers Arch. 115, 457–482 (1906).

^16^*Maxwell, S. S*.: Labyrinth and equilibrium. 1923.

^17^*Maxwell*: ibid. S. 67.

^18^It does not matter whether the cannula is inserted into the duct or the ampullar part of the transected semicircular canal. It only matters that the cupula is moved in the same direction to achieve the same effect. In the one case a suction effect, in the other case a pressure effect would be the cause for the same movement.

^19^*Breuer, J*.: Med. Jb. 1874, H. 1; 1875 H. 1.

^20^*Bethe, A*.: Biol. Zbl. 14, 579 (1894).

^21^Ampullopetal semicircular canal flow generally indicates a flow from the semicircular canal toward the ampulla into the utricle, the reverse flow is denoted as ampullofugal semicircular canal flow.

^22^*Ewald, I. R*.: Physiologische Untersuchungen über das Endorgan des Nervus octavus, S. 259. Wiesbaden 1892.

^23^*Brünings*: Verh. dtsch, otol. Ges. Dresden 1910, 192.

^24^See *A. Kreindler*: Z. Neur. 138, 699–703 (1932) und *P. Vogel*: Pflügers Arch. 230, 16–32 (1932).

^25^The deviation during normal rotatory stimulation was observed by *Steinhausen* [see Plügers Arch. 228, 322 (1931)] for the first time in freshly prepared preparations.

^26^*Gaede*: Arch. Ohr. etc. Heilk. 110, 6 (1922). *Rohrer*: See *Rohrer* u. *Masuda, Bethes* Handbuch der Physiologie, Bd. 11, S. 985–1101. 1927. *Schmaltz*: Proc. Roy. Soc. Med. sec. otol. Sitzung vom 1. Mai 1931, 374.

^27^See also *Steinhausen*: Arch. Ohr. etc. Heilk. 132, 155 (1932).

^28^*Gaede, W*.: Arch. Ohr. etc. Heilk. 110, 9 (1922).

^29^*Schmaltz, G*.: Proc. Roy. Soc. Med. sec. otol. Sitzung vom 1. Mai 1931, 374.

^30^*Schmaltz* discusses for the pure endolymph equation [Equation (2) above] (i.e., without consideration of the cupula effect) the case $\frac{d^{2}\xi }{dt^{2}}\;=\;const,$ whereby a homogeneously accelerated motion is thus assumed for the motion of the wall (*Schmaltz:* 1.c., p. 371). For constant acceleration (g = const.) the equation is as following: $m\frac{d^{2}x}{dt^{2}}\;+\;k\frac{dx}{dt}\;=\;-a^{2}x\;+\;\frac{a^{2}}{2}\;\gamma t^{2}\;+\;k\gamma t$ The solution of this equation is:

$$\begin{array}{l} x\;=\;\frac{u_{0}\;+\;x_{0}\beta_{2}\;+\;\frac{m}{a^{2}}\gamma \beta_{2}}{\beta_{2}\;-\;\beta_{1}}\;e^{-\beta_{1}t}\;-\;\frac{u_{0}\;+\;x_{0}\beta_{1}\;+\;\frac{m}{a^{2}}\gamma \beta_{1}}{\beta_{2}\;-\;\beta_{1}}\;e^{-\beta_{2}t}\;+\;\frac{1}{2}\gamma t^{2}\;\\ \;-\;\frac{m}{a^{2}}\gamma \end{array}$$

for a^2^ = 0 (i.e., without consideration of the cupula) the motion equation of the endolymph results in the equation of Schmaltz (*Schmaltz:* I.c., p. 371):

$$\begin{array}{l} m\frac{d^{2}x}{dt^{2}}\;+\;k\frac{dx}{dt}\;=\;k\gamma t\; \end{array}$$

and the solution translates into:

$$\begin{array}{l} x\;=\;\frac{1}{2}\gamma t^{2}\;+\;\gamma \frac{m^{2}}{k^{2}}\;-\;\frac{m}{k}\gamma t\;-\;\frac{m^{2}}{k^{2}}\gamma e^{-\;\frac{k}{m}t} \end{array}$$

^31^See text books on theoretic physics, e.g., *Clemens Schäfer*.

^32^Masuda: Pflügers Arch. 107, 1–65 (1922).

## References

[1] Van de WaterTR. Historical aspects of inner ear anatomy and biology that undelie the design of hearing and balance prosthetic devices. Anatomical Rec. (2012) 295:1741–59. 10.1002/ar.2259823045252

[2] CanalisRFMiraEBonandriniLHinojosaR. Antonio Scarpa and the discovery of the membranous inner ear. Otol Neurotol. (2001) 22:105–12. 10.1097/00129492-200101000-0002011314703

[3] BreuerJ. Über die function der bogengänge des ohrlabyrinths. Med Jahrbücher. (1874) 4:72–124.

[4] RetziusG. Das Gehörorgan der Wirbelthiere. Stockholm: Samson & Wallin (1881).

[5] BechterewW. Ergebnisse der Durchschneidung des N. acusticus, nebst Erörterung der Bedeutung der semicirculären Canäle für das Körpergleichgewicht. Archiv Gesamte Phys Menschen Tiere. (1883) 30:312–47. 10.1007/BF01674334

[6] BertholdE. Ueber die Function der Bogengänge des Ohrlabyrinths. Archiv Ohrenheilkunde. (1874) 9:77–95. 10.1007/BF01803958

[7] CrumBrown A. The sense of rotation and the anatomy and physiology of the semicircular canals of the internal ear. J Anat Physiol. (1874) 8(Pt 2):327–31. 17231027PMC1318976

[8] EwaldJR. Physiologische Untersuchungen üBer das Endorgan des N. Oktavus. Wiesbaden: Bergmann (1892).

[9] GoltzF. Ueber die physiologische Bedeutung der Bogengänge des Ohrlabyrinths. Archiv Gesamte Phys Menschen Tiere. (1870) 3:172–92. 10.1007/BF01855753

[10] MachE. Über den Gleichgewichtssinn. Mitt Akad Wiss Wien. (1874) 69:44–51.

[11] LeeFS. Study of the senses of equilibrium. J Physiol. (1894) 15:311–48.10.1113/jphysiol.1893.sp000475PMC151449116992097

[12] KuboI. Über die vom N. acusticus ausgelösten Augenbewegungen. II. Mitteilung: Versuche an Fischen. Pflügers Arch Physiol. (1906) 115:457–82. 10.1007/BF01677353

[13] McNallyWJTaitJ. Ablation experiments on the labyrinth of the frog. Am J Physiol. (1925) 75:155–79. 10.1152/ajplegacy.1925.75.1.155

[14] TaitJMcNallyWJ. Some features of the action of the utricular maculæ (and of the associated action of the semicircular canals) of the frog. Philos Trans R Soc London Ser B Biol Sci. (1934) 224:241–86. 10.1098/rstb.1934.0019

[15] McNallyWJTaitJ. Some results of section of particular nerve branches to the ampullae of the four vertical semicircular canals of the frog. Q J Exp Physiol. (1933) 23:147–96. 10.1113/expphysiol.1933.sp000593

[16] MowrerOH. The electrical response of the vestibular nerve during adequate stimulation. Science. (1935) 81:180–1. 10.1126/science.81.2094.180-a17817013

[17] RossDA. Electrical studies on the frog's labyrinth. J Physiol. (1936) 86:117–46. 10.1113/jphysiol.1936.sp00334816994740PMC1394590

[18] HennVYoungLR. Ernst Mach on the vestibular organ 100 years ago. Otorhinolaryngol Relat Spec. (1975) 37:138–48. 10.1159/0002752181093083

[19] WiestGBalohRW. The pioneering work of Josef Breuer on the vestibular system. Arch Neurol. (2002) 59:1647–53. 10.1001/archneur.59.10.164712374505

[20] SteinhausenW. Über Sichtbarmachung und Funktionsprüfung der Cupula terminalis in den Bogengangsanipullen des Labyrinthes. Pflüger's Archiv Gesamte Physiol Menschen Tiere. (1927) 217:747–55. 10.1007/BF01723723

[21] SteinhausenW. Über den Nachweis der Bewegung der Cupula in der intakten Bogengangsampulle des Labyrinthes. Pflüger's Archiv Gesamte Physiol Menschen Tiere. (1931) 228:807. 10.1007/BF01755546

[22] WittmaackK. Über den Tonus der Sinnesendstellen des Innenohres. Archiv Ohren Nasen Kehlkopfheilkunde. (1929) 120:256–96. 10.1007/BF01583294

[23] SteinhausenW. Über die Beobachtung der Cupula in den Bogengangsampullen des Labyrinths des lebenden Hechts. Pflügers Archiv Euro J Physiol. (1933) 232:500–12. 10.1007/BF01754806

[24] MayneR. The dynamic characteristics of the semicircular canals. J Comp Physiol Psychol. (1950) 43:309–19. 10.1037/h005482715436890

[25] Van EgmondAAGroenJJJongkeesLB. The mechanics of the semicircular canal. J Physiol. (1949) 110:1–17. 10.1113/jphysiol.1949.sp00441615406377PMC1392780

[26] GoldbergJMFernandezC. Physiology of peripheral neurons innervating semicircular canals of the squirrel monkey. 3. Variations among units in their discharge properties. J Neurophysiol. (1971) 34:676–84. 10.1152/jn.1971.34.4.6765000364

[27] GroenJJLowensteinOVendrikJH. The mechanical analysis of the responses from the end-organs of the horizontal semicircular canal in the isolated elasmobranch labyrinth. J Physiol. (1952) 117:329–46. 10.1113/jphysiol.1952.sp00475214946739PMC1392534

[28] O'LearyDPDunnRFHonrubiaV. Functional and anatomical correlation of afferent responses from the isolated semicircular canal. Nature. (1974) 251:225–7. 10.1038/251225a04424704

[29] PaulinMGHoffmanLF. Models of vestibular semicircular canal afferent neuron firing activity. J Neurophysiol. (2019) 122:2548–67. 10.1152/jn.00087.201931693427PMC6966309

[30] McLarenJWHillmanDE. Displacement of the semicircular canal cupula during sinusoidal rotation. Neuroscience. (1979) 4:2001–8. 10.1016/0306-4522(79)90071-X316878

[31] RabbittRDBrenemanKDKingCYamauchiAMBoyleRHighsteinSM. Dynamic displacement of normal and detached semicircular canal cupula. J Assoc Res Otolaryngol. (2009) 10:497–509. 10.1007/s10162-009-0174-y19513793PMC2774407

[32] OmanCMYoungLR. The physiological range of pressure difference and cupula deflections in the human semicircular canal. Theoretical considerations. Acta Otolaryngol. (1972) 74:324–31. 10.3109/000164872091284584639724

[33] YamauchiARabbittRDBoyleRHighsteinSM. Relationship between inner-ear fluid pressure and semicircular canal afferent nerve discharge. J Assoc Res Otolaryngol. (2002) 3:26–44. 10.1007/s10162001008812083722PMC3202362

[34] RabbittRDYamauchiAMBoyleRHighsteinSM. How endolymph pressure modulates semicircular canal primary afferent discharge. Ann N Y Acad Sci. (2001) 942:313–21. 10.1111/j.1749-6632.2001.tb03756.x11710473
